# CircESRP1 inhibits clear cell renal cell carcinoma progression through the CTCF-mediated positive feedback loop

**DOI:** 10.1038/s41419-021-04366-4

**Published:** 2021-11-13

**Authors:** Lin-Jing Gong, Xin-Yuan Wang, Xu-dong Yao, Xu Wu, Wen-Yu Gu

**Affiliations:** 1 Department of Respiratory and Critical Care Medicine, West China Hospital, Sichuan University, No 37 Guoxue Alley, 610041 Chengdu, Sichuan China; 2 Department of Pulmonary Medicine, Zhongshan Hospital, Fudan University, 180 Feng Lin Rd, Shanghai, 200032 China; 3 Department of Orthopaedics, West China Hospital, Sichuan University, No 37 Guoxue Alley, 610041 Chengdu, Sichuan China; 4 Department of Urology, Shanghai Tenth People’s Hospital, Tongji University School of Medicine, No. 301, Yanchang Rd., Shanghai, 200072 China

**Keywords:** RNA, Cancer epidemiology, Prognostic markers, Cancer therapy

## Abstract

Circular RNA (circRNA), a closed continuous loop formed by back-splicing, has been confirmed to be implicated in a variety of human diseases including cancers. However, the underlying molecular mechanism of circRNA regulating the progression of renal cell carcinoma (RCC) remains largely unclear. In the present study, we identified a novel circular RNA, circESRP1, that derived from the ESRP1 gene locus at 8q22.1 exons. Lower expression of circESRP1 was found in clear cell RCC (ccRCC) tissues and cell lines. Besides, circESRP1 expression level showed inversely correlated with the advanced tumor size, TNM stage and distant metastasis of ccRCC. The expression level of circESRP1 exhibited a positive correlation with CTCF protein but negatively correlated with miR-3942 in 79 ccRCC tissues. In vivo experiments, we found that overexpression of circESRP1 effectively repressed xenograft tumor growth and inhibited c-Myc-mediated EMT progression. CircESRP1 acted as a sponge to competitively bind with miR-3942 as confirmed through RNA pull-down, RIP and dual-luciferase reporter assays. Moreover, CTCF, a downstream target of miR-3942, was validated to specifically promote the circESRP1 transcript expression and regulated by circESRP1/miR-3942 pathway to form a positive feedback loop. We also revealed that the circESRP1/miR-3942/CTCF feedback loop regulated the ccRCC cell functions via c-Myc mediated EMT process. This study provides a novel regulatory model of circRNA via forming a positive-feedback loop that perpetuates the circESRP1/miR-3942/CTCF axis, suggesting that this signaling may serve as a novel target for the treatment of ccRCC.

## Introduction

Renal cell carcinoma (RCC) is one of the most common but lethal urological tumors, accounting for 2–3% of adult malignancies [1]. Generally, clear cell RCC (ccRCC) comprises 80–90% of RCC, and is reported as the most common histologic subtype with higher invasive ability and relapse rate [2]. Accumulating evidence has demonstrated ~20–30% of patients present with metastatic disease at initial diagnosis [3, 4]. Moreover, ccRCC resists radiotherapy and chemotherapy after primary radical surgery [5–7]. Thus, it is urgent to elucidate the potential molecular mechanisms underlying malignant ccRCC and determine effective therapeutic strategies.

Circular RNAs (circRNAs) are covalently closed continuous loops formed by back-splicing, most commonly derived from the exon or intron of gene precursor mRNA (pre-mRNA) [8]. Regarding the unique structures and high stability, circRNAs could bind to RNA-associated proteins and regulate transcription by competing with linear splicing [9, 10], and also act as protein decoys or miRNA sponges to assume their roles of competing endogenous RNAs (ceRNAs) [11, 12], thereby identified as important molecules in biological processes and tumor progression [13]. Recently, an increasing number of circRNAs have been reported to be associated with malignant behavior in ccRCC. For instance, Li and his colleague demonstrated that circTLK1 played a vital role in the RCC progression and patients with higher level of circTLK1 represented a poor prognosis [14]. Moreover, it has been identified circPTCH1 as a novel target for therapeutic intervention of metastatic RCC, as it could promote RCC metastasis via activating the EMT process [15]. However, only a few upregulated circRNAs have been characterized, and the suppressive phenotype of circRNAs in RCC is largely unknown.

As a ubiquitously expressed transcription factor, CCCTC-binding factor (CTCF) is reported to be associated with malignant behavior in tumors [16]. Meanwhile, it has been shown to participate more or less in the regulation of EMT pathway [17]. Recent year, some studies indicated that CTCF may affect the progression of RCC and influenced the prognosis of those patients [18]. Nevertheless, whether CTCF regulates ccRCC-relevant circRNAs and the potential molecular mechanisms remain unknown.

In the present study, the expression profiles of circRNAs in RCC tissues from GEO database were analyzed. A novel circRNA that derived from the ESRP1 gene locus at 8q22.1 exons was identified. Here, we provided a regulatory model of circRNA via circESRP1/miR-3942/CTCF axis. These findings demonstrated that the positive-feedback pathway between ‘circESRP1-CTCF’ effectively inhibited ccRCC cell growth and metastasis, suggesting the potential target for the treatment of ccRCC.

## Materials and methods

### Human tissue specimens

The pairs of snap-frozen ccRCC tissues and paired adjacent normal kidney tissues were collected between January 2015 and December 2018 for validation. Histological and pathological diagnoses of the specimens were confirmed according to the 2016 World Health Organization Consensus Classification and Staging System of Renal Tumor and Fuhrman grade by two experienced pathologists. All specimens were obtained with appropriate informed consent of the patients and approved by the Ethics Committee of Shanghai Tenth People’s Hospital of Tongji University, and written informed consent was obtained from all patients.

### GEO data

CircRNA filtration was carried out on Gene Expression Omnibus (GEO) database (GEO accession: GSE100186). A total of 4 paired ccRCC and matched non-tumor tissues were included in this circRNA expression dataset. The circRNA expression profile was calibrated and standardized using the R package at first, and then differentially expressed circRNAs were identified using the limma package with *P* < 0.05 and |log2FC|>2.

### TCGA data analysis

miRNA expression data obtained from the TCGA were used to assess the different miRNAs level in KIRC (545 cases) and normal tissues (71 cases). The data analysis was performed by the Limma R package.

### Cell lines

Human RCC cell lines 786-O, ACHN, Caki-1, A498, and normal kidney cell line HK-2 were purchased from the ATCC. All cell lines were expanded to passage 3, stored in liquid nitrogen, and used for fewer than 4 months after receipt or resuscitation from cryopreservation. Cells were cultivated in Roswell Park Memorial Institute (RPMI)-1640 medium containing 10% fetal bovine serum (Gibco, Grand Island) at 37 °C with 5% CO_2_.

### RNA-Fluorescence in situ hybridization

FISH assay was performed with Cy3-labeled circESRP1 (5′-AATAAGTTCCATCTTGCTGCACCAGCAATTTTAAGG-3′) and FITC-labeled miR-3942-5p probes (5′-ATTTCAGGTAACAGTATTGCTT-3′) synthesized by GenePharma (Shanghai, China). The FISH staining was performed using a fluorescence in situ hybridization kit (RiboBio, Guangzhou, China) according to the manufacturer’s protocol in ccRCC cells (786O, ACHN). 4′,6-diamidino-2-phenylindole (DAPI, Beyotime, China) was administrated to stain the cell nuclei, and the subcellular distributions of circESRP1 and miR-3942-5p in ccRCC cells were photographed with a confocal laser scanning microscope (Nikon, Japan).

### Sanger sequencing

RNA was extracted from RCC cells and the cDNA was reversely transcribed using the Takara PrimeScript RT Reagent Kit. Sanger sequencing was performed by the Geneseed Biotech Company (Guangzhou, China).

### Plasmid construction and cells transfection

The circESRP1-overexpressing lentivirus plasmid, miR-3942-5p mimics, and inhibitor were designed and synthesized by RiboBio (Guangzhou, China). Virus-containing supernatant was collected 48 h after lentivirus packaging, followed by its addition to the ccRCC cells. After 24 h incubation, the stably infected cells were selected with 2 μg/mL of Puromycin purchased from Selleck (Shanghai, China). CTCF small hairpin RNA (shRNA) plasmids (shCTCF-1, 5′-GCAAGGCAAGAAATGCCGTTA-3′; shCTCF-2, 5′-GCGGAAAGTGAACCCATGATA-3′) were synthesized by GenePharma (Shanghai, China). Full length of CTCF was constructed into pcDNA3.1 vector (Invitrogen, Carlsbad, USA) for overexpression of CTCF. Small‐interfering RNA (siRNA) of c-Myc (5′-GGAAACGACGAGAACAGUU-3′) was synthesized by and purchased from Genechem (Shanghai, China). The transfection assays were conducted according to the manufacturer’s protocol by using Lipofectamine 2000.

### EdU and colony formation assay

The proliferation potential of ccRCC cells was assessed using EDU and colony formation assay. For colony formation assays, 1000 cells in 2 ml medium were seeded in 6-well plates. For the EDU, the assay was conducted using EdU Apollo DNA in vitro kit (RiboBio, Guangzhou, China) following the manufacturers’ instructions. The results were detected under immunofluorescence microscope (Olympus IX73, Tokyo, Japan).

### Cell invasion and transwell assay

Cell invasion and transwell assays were performed as described previously [19, 20]. The invaded or migrated cells were counted in ten randomly microscopic fields.

### Wound healing assay

Wound healing assay was performed as previously reported [19], and the results were measured by Migration index using Image J software.

### RNA extraction and qPCR assay

Total RNAs from tissues or cells were isolated using Trizol reagent (Invitrogen), and miRNA was extracted using the SanPrep Column microRNA Extraction Kit (Sangon, Shanghai, China). Cytoplasmic and nuclear RNA was isolated using PARIS Kit (Life Technologies, ThermoFisher, Waltham, USA) according to the manufacturer’s instructions. cDNA was synthesized using PrimeScript RT Reagent (Takara) or Mir-XTM miRNA First-Strand Synthesis kit (Takara, Kusatsu, Japan). qRT-PCR was applied using a Bio-Rad CFX96 system (Bio-Rad, Foster City, CA, USA). The mRNAs and circRNAs expression in tissues or cell lines were normalized to GAPDH expression. The miRNAs expression was normalized to U6 expression. Relative RNA expression was calculated using the 2^−ΔΔCt^. The primers sequences used for qRT-PCR are shown in Table S1.

### RNase R resistance analysis of circESRP1 and actinomycin D treatment assay

The ccRCC cells were treated with or without RNase R (4 U/μg, Epicenter, Madison, USA) and incubated for 30 min at 37 °C. To study the stability of circESRP1 and linear ESRP1, the quantification of their half-lives was detected by actinomycin D treatment assay. The ccRCC cells were exposed to Actinomycin D (2 μg/mL) for 0, 4, 8, 12, or 16 h to against new RNA synthesis. The relative expression levels of RNAs were measured using qRT-PCR assay.

### Chromatin immunoprecipitation (ChIP)

To determine the cross-talking between CTCF and the ESRP1 promoter, a ChIP assay was performed according to Pierce Magnetic ChIP Kit (Thermo Scientific). Briefly, the DNA–protein complex obtained from 786O cells was sonicated to disassemble the cross-linked chromatin DNA fragments to be 200–1000 bp. Then, immunoprecipitation was implemented with 2 μg of anti-CTCF antibody (Abcam, Burlingame, CA, USA) and protein A-Sepharose beads. Meanwhile, IgG acted as a negative control. After washing the cross-links, the enriched ESRP1 promoter was purified and then analyzed by qRT-PCR with validated primers. The primer sequences were designed and synthesized by GenePharma (Shanghai, China). The qRT-PCR products were validated by agarose gel electrophoresis.

### RNA pull-down

The biotinylated 3′ end of miR-3942-5p mimic or control RNA (GenePharm Biotech) was transfected into ccRCC cells (100 nM). Two days after transfection, whole-cell lysates were harvested. The biotinylated RNA complex was pulled down by streptavidin magnetic beads at 4 °C on the rotator overnight. The abundance of circESRP1 in the bound fraction was evaluated by qRT-PCR.

### RNA immunoprecipitation (RIP) assay

The Argonaute-2 (AGO2)-RIP assay was performed using the MagnaRIP RNA-Binding Protein Immunoprecipitation Kit (Millipore, MA, USA). Briefly, ccRCC cells were harvested and lysed in RIP lysis buffer, and then incubated with magnetic beads coated with 5 μg of control rabbit IgG or antibody against AGO2 (Abcam, MA, USA) with rotation at 4 °C overnight. The immunoprecipitated RNAs were extracted as described above and detected by qRT-PCR.

### Luciferase reporter assay

The potential binding sites of miR-3942-5p and circESRP1 or CTCF were obtained from miRanda (http://www.microrna.org/) and TargetScan (http://www.targetscan.org/). Then the wild type (WT) and mutant (MUT) containing the predicted sequences were cloned into the luciferase reporter vector (Promega, Madison, WI, USA). ccRCC cells were then co-transfected with WT or MUT luciferase reporter vector with miR-3942-5p or control miRNA. For the luciferase promoter assay, the ESRP1 promoter WT or MUT luciferase reporter vector was co-transfected with CTCF plasmid or control pRL-TK Renilla plasmid into ccRCC cells. After 48 h, the firefly luciferase activities were determined comparing to Renilla using Dual-Luciferase Reporter Assay System (Promega).

### Western blot

Proteins were separated by electrophoresis and then transferred to PVDF membrane for the incubation with primary antibodies, including anti-E-cadherin (1:1000, Cell Signaling Technology, #3195), anti-N-cadherin (1:1000, Cell Signaling Technology, #13116), anti-Claudin-1 (1:1000, Cell Signaling Technology, #13255), anti-CTCF (1:1000, Cell Signaling Technology, #3418), and anti-c-Myc (1:1000, Cell Signaling Technology, #9402). GAPDH (1:500, Beyotime Biotechnology, AF1186) acted as the internal control. After incubation overnight at 4 °C, the immunoreactive blots were washed and administrated with HRP-conjugated secondary antibodies at room temperature for 1 h. Finally, the blots were detected by ECL (Beyotime Biotechnology).

### In vivo xenograft experiment

The animal experiment was approved by the Ethics Committee of Shanghai Tenth People’s Hospital of Tongji University. ACHN cell line stably expressing firefly luciferase was constructed. The male BALB/c nude mice (6- to 8-week old) were purchased and randomly divided into two groups (*n* = 4 per group) for subcutaneously injection of 1 × 10^6^ Luc-ACHN cells (Vector or OE-circESRP1). Tumor progress was observed using the IVIS imaging system (PerkinElmer, USA) for once a week. Subsequently, the mice were sacrificed 5 weeks later. The harvested organs were immediately excised and weighted. Last, these tumors were assessed for IHC staining and PCR assay.

### In vivo metastatic model

The male BALB/c nude mice (6- to 8-week old) were cared for based on our institution’s protocols for ethical animal care, and the experiment was approved by the Ethics Committee of Shanghai Tenth People’s Hospital of Tongji University. Mice were randomly assigned into two groups, control and experimental groups (*n* = 6 per group). A total of 1 × 10^6^ ACHN cells (Vector or OE-circESRP1) were suspended in 100 μl of sterile PBS and administrated to mice through tail vein injections. Then, all mice were sacrificed for histological examination of lung metastases after 4 weeks of tail vein injections.

### Immunohistochemistry (IHC)

Tumor tissues of patients or mice were fixed in 10% (v/v) formaldehyde in PBS, embedded in paraffin, and cut into 4-μm sections and used for IHC staining with specific primary antibodies against PCNA (1:2000, Cell Signaling Technology), c-Myc (1:500, Abcam), Vimentin (1:200, Cell Signaling Technology), E-cadherin (1:400, Cell Signaling Technology), and CTCF (1:1000, Cell Signaling Technology). The representative images were acquired under a light microscope (Olympus IX73, Tokyo, Japan). Positive degree was calculated as the number of immunopositive cells × 100% divided by the total number of cells/field in 10 random fields at ×400 magnification.

### Statistical analysis

All experiments were performed at least three times. The data were presented as means ± SEM. Statistical analyses were conducted using SPSS software (Version 25.0) and GraphPad Prism (Version 7.0). Group differences were tested for statistical significance using Student’s *t*-test (two-tailed), one-way analysis of variance (ANOVA), Chi-square test (the frequency test), and Spearman’s correlation, as appropriate. *P* < 0.05 was considered statistically significant.

## Results

### circESRP1 is lowly expressed in the RCC tissue and cells

**Fig. 1:**
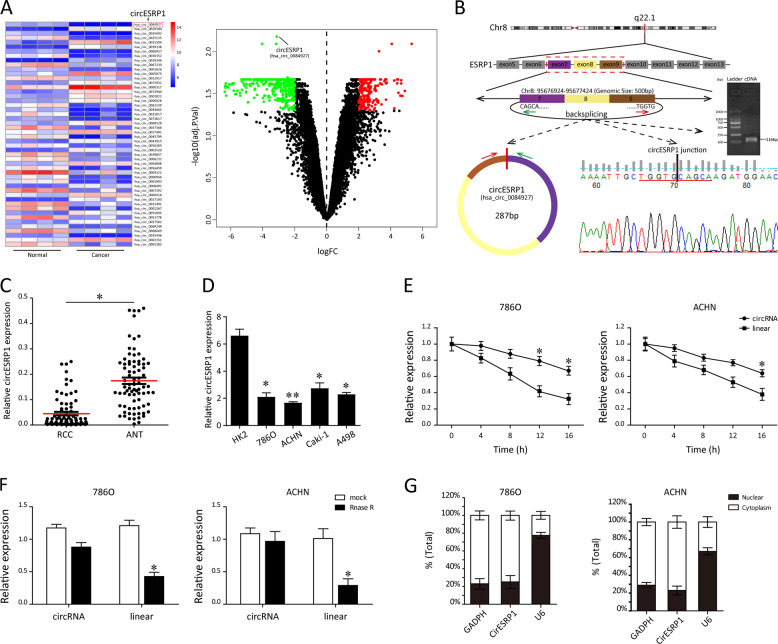
circESRP1 is downregulated in the ccRCC tissues and cells. **A** The heatmap and volcano plot of circRNA profiles in RCC and normal tissues in the dataset from the GEO database (GSE100186). The variation in RCC-related differentially expressed circRNAs from GSE65071 was compared and illustrated. **B** Schematic diagram illustrated the formation of circESRP1. The RT-PCR product and back-splicing junction of circESRP1 were validated by agarose gel electrophoresis and Sanger sequencing. **C** qRT-PCR analysis showed the expression levels of circESRP1 in ccRCC tissues were significantly lower than that in adjacent normal kidney tissues. **D** Relative expression fold changes of circESRP1 in ccRCC cell lines were screened by qRT-PCR. 786O, ACHN, Caki-1, and A498 exhibited lower circESRP1 expression, while HK2 was circESRP1-high-expressed. **E** The transcript half-life of circESRP1 and linear ESRP1 in 786O and ACHN cells treated with transcription inhibitor Actinomycin D was analyzed by qRT-PCR. **F** RNase R was administrated to the extracted RNA to detect the relative expression of circular form (circESRP1) and the linear form (ESRP1 mRNA). **G** Levels of circESRP1 in the nuclear and cytoplasmic fractions of 786O and ACHN cells. Data are the means ± SEM of three independent experiments. *P < 0.05; **P < 0.01.

In this study, we first analyzed the circRNA gene chip GSE100186 from GEO database (https://www.ncbi.nlm.nih.gov/geo/). Among these differentially expressed circRNAs, we focused on circESRP1, which was identified to be aberrantly lowly expressed in RCC cancer (Fig. 1A). Previous study has demonstrated that circESRP1 served as a vital biomarker of chemosensitivity in small cell lung cancer (SCLC) patients [21]. However, the potential functions in RCC patients remained unknown. As shown in Fig. 1B, circESRP1 was derived from the ESRP1 gene (chr8:95676924–95677424) and generated from its exons 7, 8, and 9 via back-splicing. After predicting the sequence of circESRP1 back-splicing junction on circBase (http://www.circbase.org/), we designed a specific primer and validated the back-splicing junction of circESRP1 by Sanger sequencing. The presence of circESRP1 was also proved by qRT-PCR and agarose gel electrophoresis. Overall, we made the validation of characterization for circESRP1 in RCC tissue and cells. In line with previous finding, qRT-PCR analysis suggested circESRP1 expression was notably decreased in 79 paired RCC tissues compared with adjacent non-tumor tissues (ANT, Fig. 1C). Similar findings were also observed in ccRCC and HK2 cell lines analyzed by qRT-PCR, as shown in Fig. 1D. To further verify the stability of circESRP1 in ccRCC cells (786O, ACHN), we treated cells with Actinomycin D or RNase R, respectively. After the ccRCC cells were treated with Actinomycin D, circESRP1 exhibited an obviously longer half-life than did linear transcript (Fig. 1E). Moreover, as illustrated in Fig. 1F, circESRP1 was able to be more resistant to RNase R degradation, suggesting the circular structure of circESRP1 in ccRCC cells. Next, the subcellular localization of circESRP1 was assessed in ccRCC cells (786O, ACHN) by nuclear-cytoplasmic fractionation assay. The results showed that circESRP1 transcripts were located predominantly in the cytoplasm (Fig. 1G).

### Overexpression of circESRP1 inhibits RCC progression in vitro

**Fig. 2:**
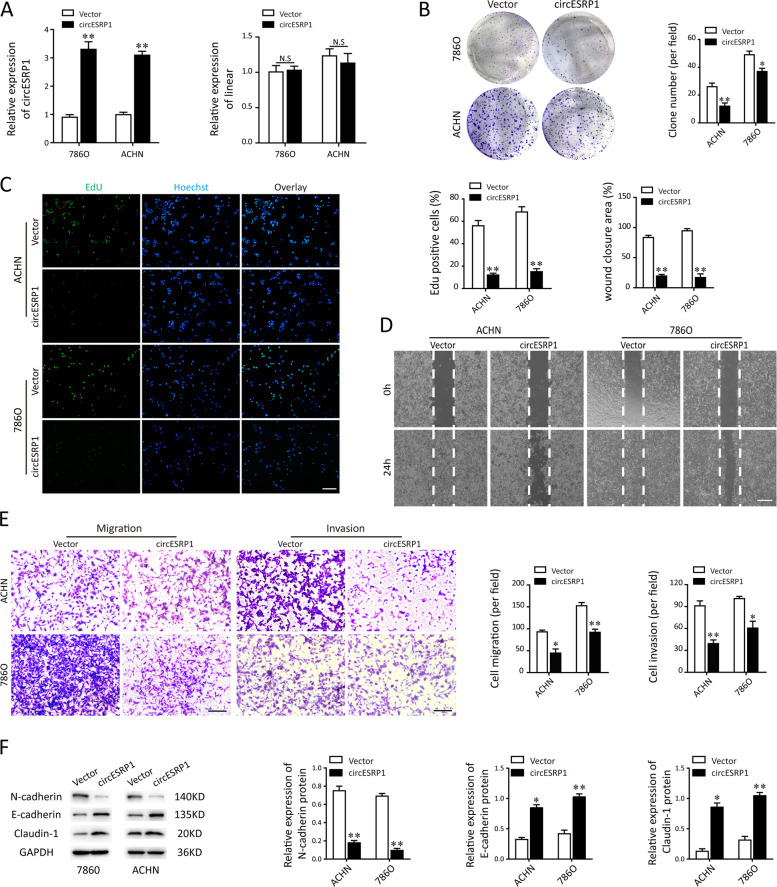
Overexpression of circESRP1 suppressed renal cancer progression. **A** ccRCC cells that stably overexpressed circESRP1 were constructed. The circESRP1 and linear ESRP1 mRNA level were measured using qRT-PCR. **B** Colony formation assay was performed in both 786O and ACHN cell lines after overexpression with circESRP1. **C** Cell proliferation rate in ccRCC cells (786O, ACHN) under the specific conditions was performed by EdU assay. Scale bar: 100 μm. **D**, **E** The biological role of circESRP1 on cell migration and invasion capability was assessed by wound healing assay (**D**, scale bar: 200 μm), transwell migration assay, and matrigel invasion assay (**E**, scale bar: 50 μm) and in 786O and ACHN cells, respectively. **F** The protein markers of EMT, including mesenchymal markers (N-cadherin) and the epithelial marker (E-cadherin, Claudin-1), were measured by western blot analysis. Data are the means ± SEM of three independent experiments. *p < 0.05; **p < 0.01.

To determine the biological function of circESRP1, circESRP1-overexpressing lentivirus plasmid (OE-circESRP1) and the control vector (Vector) were transfected to ccRCC cells (786O and ACHN). After gaining the stable cell strains, the results of qRT-PCR presented that the expression of linear ESRP1 mRNA were not influenced by the OE-circESRP1 transfection (Fig. 2A). Then, we performed the colony formation assay, EdU assay, transwell assay, and wound healing assay to identify the potential antioncogenic roles of circESRP1 in vitro. Capabilities of proliferation, migration, and invasion of ccRCC cells were notably attenuated after overexpression of circESRP1, while it was higher in the circESRP1 empty control vector group (Fig. 2B–E). Subsequently, we further tested the protein markers of epithelial-mesenchymal transition (EMT) by western blot analysis. According to the results from Fig. 2F, immunoblotting showed that overexpression of cricESRP1 significantly enhanced the expression of epithelial markers (E-cadherin, Claudin-1), while inhibited the levels of mesenchymal markers (N-cadherin). Taken together, these data supported that upregulation of circESRP1 exhibited antioncogenic effects on RCC tumorigenesis in vitro.

### CTCF binds to the ESRP1 promoter and enhances circESRP1 expression

**Fig. 3:**
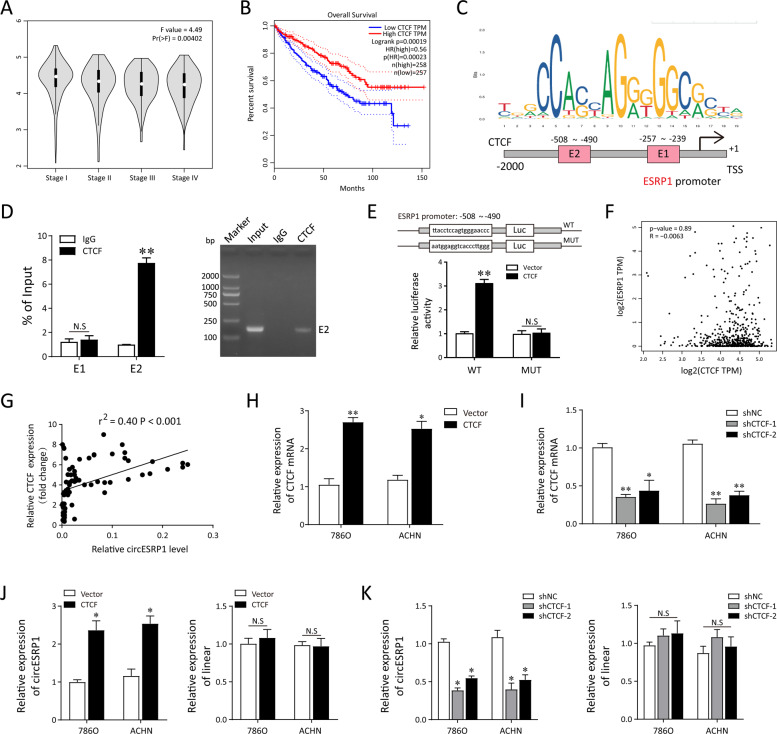
CTCF accelerates the biogenesis of circESRP1 rather than the linear mRNA of ESRP1. **A** Correlation between CTCF Expression and Tumor Stage in clear cell renal cell carcinoma Patients (GEPIA, http://gepia.cancer-pku.cn/). **B** The Prognostic Value of mRNA Level of CTCF in clear cell renal cell carcinoma Patients (GEPIA). **C** JASPAR (http://jaspar.genereg.net/) indicated that CTCF shared with the binding sites with the promoter region of ESRP1. **D** CHIP-PCR was performed with CTCF antibody in 786O cells to detect the enrichment of potential binding sequences of ESRP1 promoter region. The qRT-PCR product was validated by agarose gel electrophoresis. **E** Luciferase reporter assay showed that CTCF bound to E2 wild type rather than mutant type. **F** No significant correlation between ESRP1 level and CTCF level in clear cell renal cell carcinoma Patients based on the TCGA (GEPIA). **G** Correlation between CTCF expression and circESRP1 level in our RCC clinical samples (*n* = 79). **H** Stable overexpression plasmid for CTCF was transfected into ccRCC cells (786O, ACHN) to increase CTCF expression. The CTCF mRNA level was detected by qRT-PCR. **I** Stable silencing shRNA were transfected into ccRCC cells (786O, ACHN) to silence CTCF expression. The level of CTCF in 786O and ACHN cells was determined by qRT-PCR. **J**, **K** RT-PCR was performed to detect the expression of circESRP1 and linear ESRP1 in 786O and ACHN cells transfected with oe-CTCF, shCTCF, and control. Data are the means ± SEM of three independent experiments. *p < 0.05; **p < 0.01; N.S, not significant.

After identified the role of circESRP1 in ccRCC progression, we wondered how circESRP1 was regulated during the pathophysiological process of ccRCC tumorigenesis. CCCTC-binding factor (CTCF) is a vital zinc finger transcription factor that participating in transcription regulation [22, 23]. We found that CTCF was predicted to be able to bind the promoter region of cricESRP1 based on TRCirc (http://www.licpathway.net/TRCirc/view/index) [24]. According the public GEPIA dataset based on the TCGA (http://gepia.cancer-pku.cn/), the bivariate correlation analysis revealed a remarkable negative correlation between the CTCF mRNA expression and tumor stage in ccRCC Patients (Fig. 3A). Besides, patients with lower CTCF expression had poor overall survival (Fig. 3B). Therefore, we speculated that CTCF might mediate the biological function of RCC cells by promoting circESRP1 transcription. The bioinformatics tools predicted on JASPAR (http://jaspar.genereg.net/) and PROMO 3.0 website validated that CTCF shared with the binding sites with the promoter region of ESRP1 (Fig. 3C). Simultaneously, targeting the two potential sites, the ChIP-PCR and agarose gel electrophoresis assays were performed. The result demonstrated that the second site (−257 to −239) might effectively combine with the CTCF antibody (Fig. 3D). Furthermore, the WT and MUT sequences of ESRP1 promoter region (−257 to −239) targeting the second predicted sites were constructed, and the results of luciferase reporter assay proved the WT rather than MUT of this region could combine with the CTCF, suggesting the CTCF-mediated activation for ESRP1 transcription (Fig. 3E).

The biogenesis of circular RNAs or linear ones can derive from the same pre-mRNA through the competition between back-splicing and canonical splicing [25]. Hence, we further investigated the relationships between the CTCF expression and circESRP1, or ESRP1 mRNA levels. As shown in Fig. 3F, there was no significant correlation between CTCF and ESRP1 in the KIRC tissues from GEPIA dataset (*p* = 0.89). Nevertheless, in the enrolled RCC patients, we found that the circESRP1 expression was positively correlated with CTCF (*p* < 0.001, Fig. 3G). The expression of ESRP1 linear RNA showed no significant correlation with the expression of circESRP1 (*p* = 0.7202, Fig. S1). Next, 786O and ACHN cells were transfected with the shRNAs or pcDNA-CTCF before the assays. The transfection efficiency was examined by qRT-PCR (Fig. 3H, I) and western blot (Fig. S2). Similarly, CTCF overexpressed plasmid enhanced ESRP1 circular levels rather than ESRP1 mRNA, and CTCF silencing shRNA reduced circESRP1 level rather than linear ESRP1 in both 786O ACHN cells (Fig. 3J, K). Taken together, our results indicated that CTCF specifically regulated the expression of circESRP1 instead of ESRP1 mRNA.

### miR-3942 functions as the bridge of circESRP1/CTCF axis

**Fig. 4:**
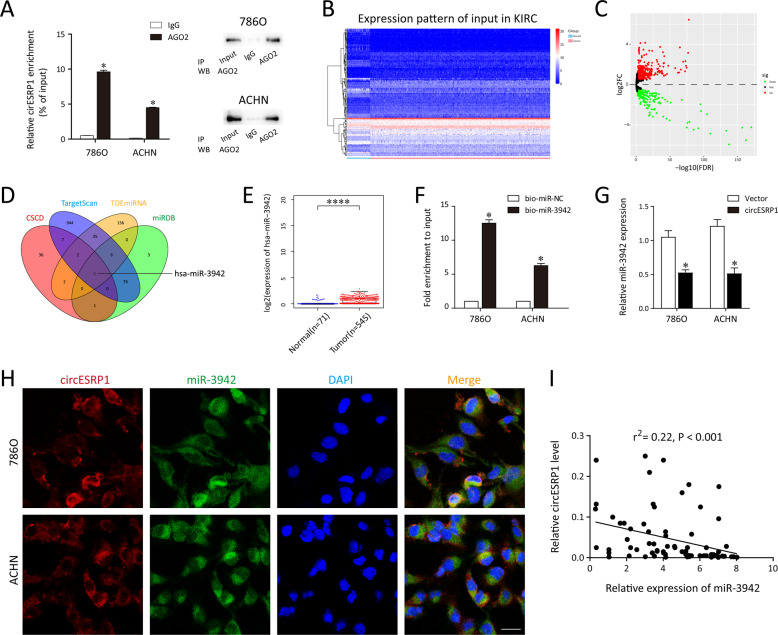
circESRP1 functions as a sponge of miR-3942 in ccRCC cells. **A** RNA immunoprecipitation (RIP) analysis of circESRP1 in 786O and ACHN cells was performed using antibody against AGO2. Western blotting analysis of immunoprecipitated AGO2 protein was shown. **B**, **C** Clustered heatmap and volcano plot of the differentially expressed miRNAs in TCGA. **D** Venn diagram showed the overlapping of target miRNAs of circESRP1 based on TargetScan (http://www.targetscan.org), miRDB (http://mirdb.org/), CSCD (http://gb.whu.edu.cn/CSCD/), and downregulated miRNA expression in TCGA results. **E** The differential expression of miR-3942 between KIRC (kidney renal clear cell carcinoma) tissue samples (*n* = 545) and normal tissue samples (*n* = 71) in TCGA. **F** Enrichment of circESRP1 in 786O and ACHN cells after pull-down assay with biotinylated miR-3942. **G** After ccRCC cells (786O, ACHN) were transfected with circESRP1-overexpressing lentivirus plasmid, the expressions of miR-3942 were detected by qRT-PCR. **H** The co-localization of miR-3942-5p and circESRP1 was observed by RNA in situ hybridization in 786O and ACHN cells. Nuclei were stained with DAPI. Bar = 100 μm. **I** Correlation between relative circESRP1 expression and relative miR-3942 expression was analyzed with Spearman’s analysis in 79 RCC clinical samples. *p < 0.05; ****p < 0.0001.

Next, we wondered whether circESRP1 could regulate the expression levels of CTCF to form a closed-loop regulation relationship. Recent studies have reported that circRNAs can function as miRNA sponges to regulate gene expression [26, 27]. RNA immunoprecipitation (RIP) analysis revealed that circESRP1 was significantly enriched by the AGO2 antibody (Fig. 4A, *P* < 0.05), suggesting that circESRP1 may act as a ceRNA in the pathogenic process of RCC. To explore the candidate miRNAs that contained putative targeting regions for CTCF, 1078 miRNAs were selected from the prediction results through TargetScan (http://www.targetscan.org/) and miRDB (http://mirdb.org/). Meanwhile, online bioinformatics tool CSCD (http://gb.whu.edu.cn/CSCD/) indicated that there were 49 miRNAs with potential binding sites for circESRP1. Subsequently, we conducted a miRNA sequencing data analysis to evaluate the miRNA expression profiles of RCC downloaded from TCGA (Fig. 4B, C). The results revealed 316 differentially expressed miRNAs in the 71 normal and 545 RCC tissues (|fold changes| ≥1 and FDR < 0.05, Fig. 4D). Finally, the intersection between bioinformatic algorithms and upregulated miRNAs in TCGA datasets identified that miR-3942 might act as the target of circESRP1 and also highly expressed in ccRCC tissues (Fig. 4D, E). As expected, pull-down assays revealed significantly higher circESRP1 enrichment by biotin-miR-3942 (Fig. 4F, *P* < 0.05). Besides, the expression of miR-3942 was remarkably decreased after 786O and ACHN cells that overexpressed circESRP1 (Fig. 4G). To observe the cellular distribution of circESRP1 and miR-3942-5p, double RNA-FISH assay was performed and indicated the co-localization of circESRP1 and miR-3942-5p in the cytoplasm (Fig. 4H). Furthermore, circESRP1 was validated to be negatively correlated with the miR-3942 in RCC individuals (Fig. 4I). In conclusion, the findings in this part manifested that circESRP1 may positive feedback regulate CTCF expression via serving as a sponge of miR-3942.

### circESRP1 acts as a sponge of miR-3942 to regulate CTCF expression

**Fig. 5:**
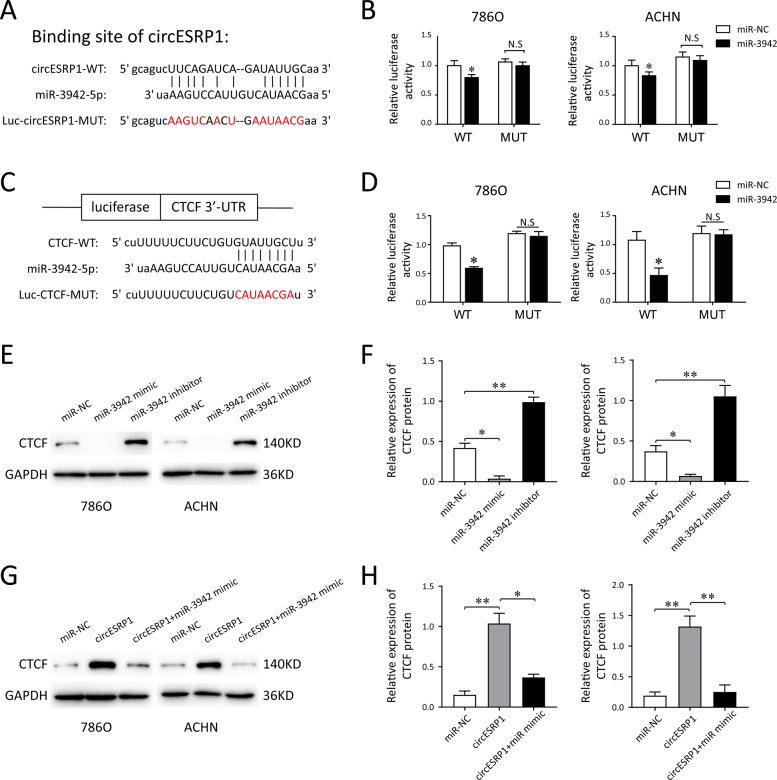
circESRP1 sponges miR-3942 to regulate CTCF expression. **A** Schematic of the predicted miR-3942 binding site on circESRP1. The circESRP1 wild-type (WT) and mutant (Mut) luciferase reporter vectors were constructed. **B** Luciferase activity of wild type or mutated circESRP1 in 786O and ACHN cells after co-transfection with miR-3942 mimic or miRNA control. **C** The CTCF wild type and mutant sequence at 3′-UTR and miR-3942-5p were constructed. **D** Luciferase reporter assays showed that co-transfected miR-3942-5p mimics significantly inhibited luciferase activity of CTCF wild type. **E**, **F** The CTCF protein levels in ccRCC cells (786O, ACHN) were assessed by western blot after transfection with miR-3942-5p inhibitors, NC or mimics. **G**, **H** Western blot analysis showed the CTCF protein in the transfection of overexpressed circESRP1 with miR-3942-5p mimics. The data are presented as the mean (*n* = 3) ± SEM. *p < 0.05; **p < 0.01.

To further determine the miRNA sponge ability of circESRP1, wild-type or mutant sequences corresponding with the miR-3942 were constructed (Fig. 5A). After co-transfection of miR-3942 mimic and circESRP1 wild type luciferase reporters into ccRCC cells, the luciferase activity was significantly reduced by ~35%, indicative of the directly binding capability of miR-3942 to circESRP1 (Fig. 5B). Given that the publicly available algorithms (TargetScan, miRDB) identified CTCF as a target of miR-3942-5p, the wild-type and mutant luciferase reporter plasmid of CTCF 3′UTR were constructed to perform luciferase reporter assay (Fig. 5C). According to the results, luciferase reporter assay validated the molecular binding for miR-3942-5p with CTCF (Fig. 5D). To further explore the interaction within CTCF and miR-3942-5p, we transfected the miR-3942-5p mimics and inhibitor into the ccRCC cells using Lipofectamine 2000. The transfection effectiveness of miR-3942 mimic and inhibitor were verified by qRT-PCR (Fig. S2C). Western blot analysis illustrated that miR-3942 mimic transfection repressed the CTCF protein, while the transfection of miR-3942 inhibitor markedly upregulated the protein level of CTCF (Fig. 5E, F). Therefore, these data support that CTCF expression level was negatively regulated by miR-3942-5p and functioned as the target of miR-3942 in ccRCC cells. We further determined whether the circESRP1 might involve in RCC tumor progression through circRNA–miRNA–mRNA signal pathway. For ccRCC cells, the transfection of OE-circESRP1 enhanced the expression of CTCF, while the co-transfection of miR-3942 mimic neutralized it (Fig. 5G, H). Taken together, these findings indicated that circESRP1 and miR-3942 had opposite effects on the CTCF expression. In addition, circESRP1 inhibited RCC progression, at least in part through serving as a miR-3942 sponge to upregulate CTCF.

### circESRP1 inhibits EMT by enhancing the suppressive effects of CTCF on c-Myc expression

**Fig. 6:**
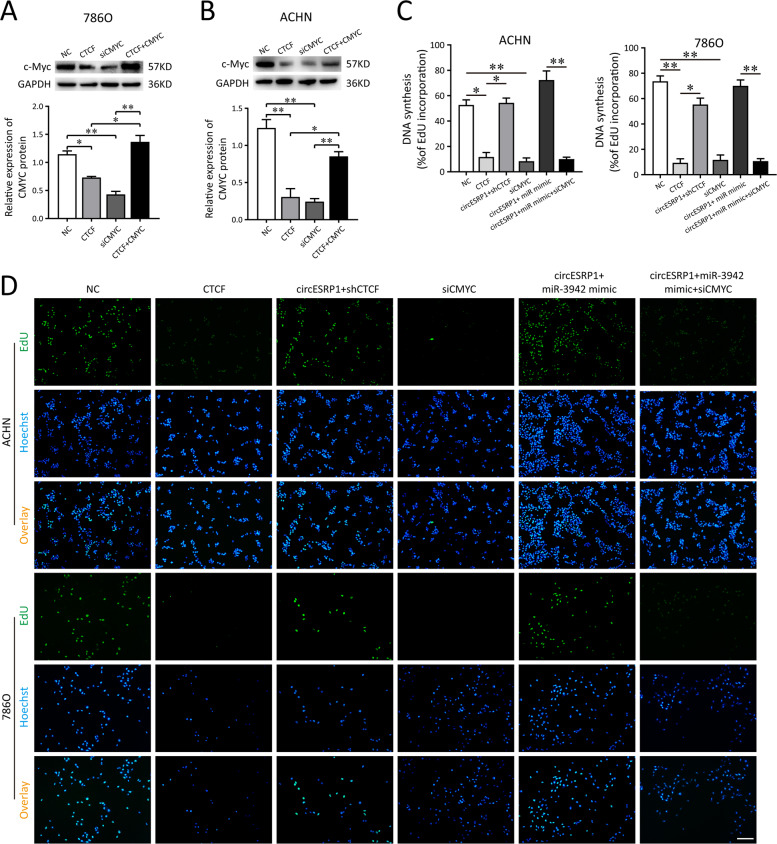
circESRP1 prohibits ccRCC proliferation via miR-3942/CTCF axis and c-Myc. **A**, **B** Western blot analysis showed the c-Myc protein level in the transfection of CTCF overexpressed plasmid with or without CMYC overexpression in ccRCC cells (786O, ACHN). **C**, **D** The DNA synthesis of 786O and ACHN cells was tested by EdU assay with the transfection of CTCF overexpressed plasmid (CTCF), CTCF shRNA (shCTCF), circESRP1-overexpressing lentivirus plasmid (circESRP1), c-Myc siRNA (si-c-Myc), and miR-3942 mimic. The data are presented as the mean (*n* = 3) ± SEM. *p < 0.05; **p < 0.01.

**Fig. 7:**
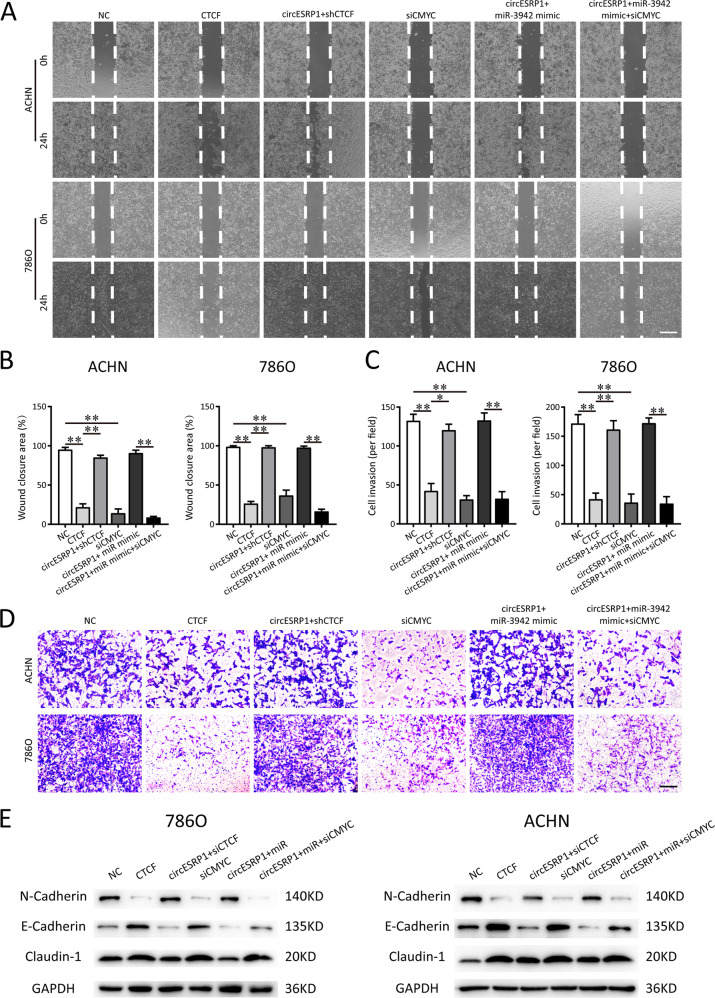
circESRP1/miR-3942/CTCF positive feedback loop modulates ccRCC metastasis. **A**, **B** The migrative ability of 786O and ACHN cells was detected using wound healing assay with the transfection of CTCF overexpressed plasmid (CTCF), CTCF shRNA (shCTCF), circESRP1-overexpressing lentivirus plasmid (circESRP1), c-Myc siRNA (si-c-Myc), and miR-3942 mimic. Scale bar: 200 μm. **C**, **D** The invasive ability of 786O and ACHN cells was detected by matrigel invasion assay. Scale bar: 50 μm. **E** Western blot showed expression of different epithelial and mesenchymal markers (N-cadherin, E-cadherin, and Claudin-1). The data are presented as the mean (*n* = 3) ± SEM. *p < 0.05; **p < 0.01.

EMT can increase malignant potential and aggressiveness of RCC. It has been confirmed that c-Myc is a transcription factor for the EMT progression in RCC [28, 29]. As CTCF was initially identified as a transcriptional repressor of the c-Myc gene [30], we speculated that interaction of CTCF with circESRP1/miR-3942 might modulate c-Myc expression and further affected the tumor phenotypes of RCC. In ccRCC cells (786O, ACHN), the pcDNA-CTCF or c-Myc siRNA transfection repressed the protein level of c-Myc. Interestingly, after the co-transfection of c-Myc overexpressed plasmid with pcDNA-CTCF, the decreased c-Myc protein level can be significantly recovered (Figs. 6A, B, S2D-E). Therefore, these findings proved CTCF could negatively regulate the c-Myc expression in ccRCC cells. Subsequently, rescue experiments were performed to identify the role of circESRP1/miR-3942/CTCF pathway and c-Myc-mediated EMT process. The transfection of pcDNA-CTCF or c-Myc siRNA could markedly inhibit the proliferation rate of 786O and ACHN cells compared with negative control (NC) group. The results of our study showed that overexpression of circESRP1 repressed the tumor phenotypes of RCC in vitro, however, the co-transfection of shCTCF or miR-3942 mimic can enhance the proliferation rate of ccRCC cells. In addition, the deficiency of c-Myc caused a significant reduction in cell proliferous abilities in most situations (Fig. 6C, D). Next, we performed wound healing assay (Fig. 7A, B), transwell invasion assay (Fig. 7C, D), and EMT marker assessment (Fig. 7E) for RCC malignant phenotype. Consistently, the cells transfected with pcDNA-CTCF and c-Myc siRNA showed lower migratory abilities, invasive abilities, and EMT characteristics compared with NC group (Fig. 7). The circESRP1 overexpression could rescue these tumor behaviors, while the co-transfection of shCTCF or miR-3942 mimic accelerated the migration, invasion, and EMT characteristics. Also, the deficient c-Myc expression could restrain the phenotype upon miR-3942 overexpression. Hence, these data identified the role of circESRP1/miR-3942/CTCF axis and c-Myc-mediated EMT process for RCC tumor behavior.

### Overexpression of circESRP1 restricts the RCC tumor growth, metastasis, and EMT markers expression in vivo

**Fig. 8:**
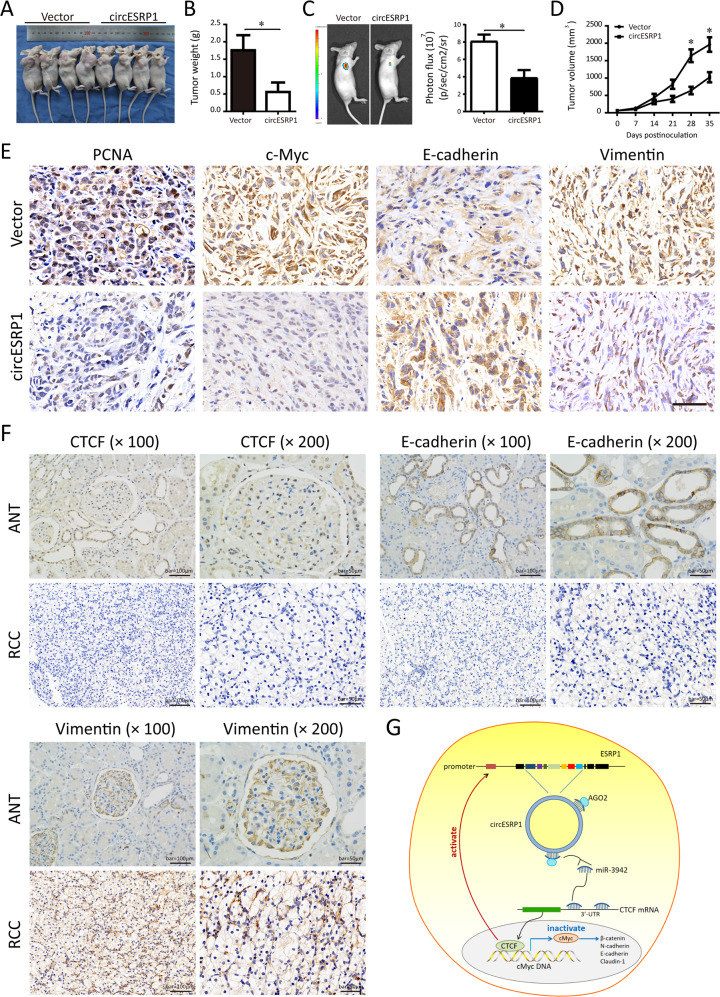
Overexpression of circESRP1 inhibits the tumor growth and EMT signaling pathway of clear cell renal cell carcinoma (ccRCC) in vivo. **A** Overexpressed circESRP1 ACHN cells were injected subcutaneously to detect in vivo effect of circESRP1. **B** The tumor weight in circESRP1 overexpressed and control group. **C** Representative bioluminescence images of mice bearing ACHN cells were obtained. **D** Effect of circESRP1 expression level on ccRCC tumor volume in xenograft tumor mice. **E** Immunohistochemical staining of the tumor tissues showed that circESRP1 overexpression inhibited the tumor phenotypes of RCC. Bar = 50 μm. **F** Immunohistochemical staining for CTCF, E-cadherin, and Vimentin in ccRCC tissues and adjacent non-tumor tissues was shown. **G** A Schematic Diagram of circESRP1/miR-3942/CTCF feedback loop in ccRCC cells EMT. *p < 0.05; **p < 0.01.

The stably overexpressed circESRP1 that labeled with firefly luciferase in ACHN cells was conducted for the xenograft. As shown in Fig. 8A–D, in vivo studies of nude mice indicated that circESRP1 overexpression significantly decreased the tumor volume and weight. Similar results were obtained using the bioluminescence in vivo imaging system. Then, the relative expressions of circESRP1, miR-3942, and CTCF were determined by qRT-PCR in Fig. S3, suggesting the presence of correlation in vivo. Furthermore, IHC staining revealed that intratumoral injection of overexpressed circESRP1 enhanced the E-cadherin expression, but decreased the levels of PCNA, c-Myc, and Vimentin, indicative of lower aggressive capabilities of tumor growth and metastasis (Fig. 8E). To further access the role of circESRP1 in ccRCC metastasis in vivo, mice were intravenously injected with ACHN cells. Overexpression of circESRP1 markedly increased pulmonary metastatic nodules compared with control group (Fig. S4). Taken together, these results indicated that circESRP1 played a critical role in RCC tumor growth and metastasis in vivo.

### Lower circESRP1 expression in RCC tissues predicts a poor prognosis

To investigate the correlation between circESRP1 expression and clinic-pathological features, we divided enrolled individuals into two groups based on circESRP1 expression value. Chi-square test was performed to analyze clinical characters between the two groups. As shown in Table 1, circESRP1 level was correlated with tumor size (*p* = 0.002), distant metastasis (*p* = 0.001) and TNM stage (*p* = 0.001), but not with age (*p* = 0.077), sex (*p* = 0.079), or ISUP grade (*p* = 0.077). It suggested that high circESRP1 expression was associated with favorable clinical outcomes. Compared to the ANT tissues, the CTCF and E-cadherin stainings were weak, while Vimentin expression was robust in the ccRCC tissues (Fig. 8F). Besides, we noticed the obvious positive correlations between the levels of circESRP1 and E-cadherin expression in ccRCC tissues, but the negative correlation existed between levels of circESRP1 and Vimentin expression (Fig. S5). Based on these findings, we proposed that circESRP1/miR-3942/CTCF positive feedback loop negatively affected the c-Myc-EMT signaling pathway in ccRCC tissues, thereby impacting the tumor behaviors of RCC (Fig. 8G).

**Table 1:** Correlation within circESRP1 expression and the clinicopathological characteristic of ccRCC patients.

Characteristics	circESRP1 expression		*P*-value
	Low (*n* = 41)	High (*n* = 38)	
Age (yrs)			0.077
≤60	14	17	
>60	27	21	
Sex (*n*)			0.079
Male	13	16	
Female	28	22	
Tumor size (cm)			*0.002*
≤4	22	28	
>4	19	10	
ISUP grade			0.077
I or II	30	23	
III or IV	11	15	
AJCC stage			*0.001*
I or II	23	29	
III or IV	18	9	
Distant metastasis			*0.001*
No	34	37	
Yes	7	1	

*P*-value <0.05 represents statistical differences.

## Discussion

The current study highlights the prognostic and therapeutic importance of circESRP1 in RCC tumorigenesis. Clinically, this termed circESRP1 was significantly downregulated in RCC tissues, thereby inversely correlated with the advanced TNM stage of ccRCC. In vivo studies of nude mice showed overexpression of circESRP1 effectively repressed xenograft tumor development and pulmonary metastasis, and inhibited c-Myc-mediated EMT progression by maintaining the low expression level of CTCF. Several circRNAs have been identified to be significantly dysregulated in RCC [31, 32]. In our research, we discovered that the ectopic lowly expressed circESRP1 was closely correlated with the poor prognosis of RCC patients.

As regarding the biogenesis of circRNAs, existing studies have demonstrated that multiple transcription factors could regulate the biogenesis of circRNA from the precursor-mRNA [33]. CTCF is proposed as a tumor suppressor in multiple cancers [34], thereby our qRT-PCR assay revealed its downregulation in RCC cell lines and tissues. The dominating circRNAs are generated from the exons of genes which are also transcribed to linear mRNA [8]. Interestingly, previous studies also indicated that not all the expression of circRNAs are dependent of related linear isoforms and some of circRNAs are more abundant than their linear transcripts [35]. In the mechanical investigation, ChIP-PCR and dual-luciferase reporter assays confirmed an interaction between transcription factor CTCF and the promoter region of ESRP1. Moreover, overexpression of CTCF significantly elevated the expression level of circESRP1, while the ESRP1 mRNA expression did not change significantly. As a result, our data revealed that CTCF could directly bind to the ESRP1 promoter and promote its transcription, then accelerating the circularization of circESRP1. However, in this case, it remains unclear how CTCF could specifically promotes the expression of circESRP1 but not linear RNA. It is speculated that this may be because ESRP1 is also an important splicing factor [20, 36, 37] or circRNA has higher stability [8, 35]. Recent years, convincing evidences has also linked CTCF to modulation of alternative splicing (AS) at both the transcriptome-wide level and individual transcript [38, 39]. Besides, Meng et al. also found that Twist1 could bind the Cul2 promoter to activate its transcription and to selectively promote expression of Cul2 circular RNA (circ-10720), but not mRNA in hepatocellular carcinoma [33]. They also indicated previous study found that some cassette exons are more favorably included in circRNAs than in linear mRNAs [40], may be the cause of this phenomenon. Anyhow, this is a very interesting finding that deserves further study in the future.

Some dysregulated circRNAs can absorb miRNA to modulate the expression of oncogenes or tumor suppressor genes in carcinogenesis and cancer development [27]. Thus, the cytoplasmic location of circESRP1 indicated the potential post-transcriptional regulation. Accumulation evidence showed that more and more non-coding RNA-associated positive feedback loops have been excavated to be closely related to tumorigenesis and progression [20, 21]. As the CTCF expression was positive correlated with circESRP1 level, we were curious whether CTCF/circESRP1 pathway was also involved in this molecular regulatory mode. GEO and bioinformatics analysis were subsequently applied to screen the target genes of circESRP1/CTCF. According to the results, we finally identified miR-3942 as one of the leading candidates. A previous study showed that miR-3942-5p overexpression promoted the disease progression [41]. Our results indicated that circESRP1 upregulated CTCF expression by relieving the post-transcriptional suppression capabilities of miR-3942, suggesting a positive feedback loop between CTCF and circESRP1/miR-3942 pathway in ccRCC. Functional experiments presented that circESRP1 or CTCF overexpression could inhibit RCC cell migration and invasion, and this effect was attenuated by a miR-3942 mimic. These data revealed the vital role of the circESRP1/miR-3942/CTCF axis in ccRCC.

As a critical oncogene, c-Myc and its downstream targets had been extensively studied in a variety of cancer types. CircESRP1 overexpression impaired the stimulating effects of CTCF knockdown on c-Myc and EMT. Previous studies proved that CTCF could interact with c-Myc promoter elements and represses its transcription [30, 37], thus the constructing of circESRP1/miR-3942-5p/CTCF feedback loop could finally inhibit RCC EMT progression via restricting the expression of c-Myc. Previous studies indicated that CTCF was a master regulator of gene expression, and the functions of which were complicated. It can function as a transcriptional activator and repressor, as well as a chromatin insulator etc [37]. Both Yang et al. [42] and Vostrov et al. [43] demonstrated that CTCF can bind the promoter of the amyloid b-protein precursor (APP) gene and promoted its transcriptional activation. Also, some studies showed that CTCF was associated with the origin and evolution of the non-coding regulatory genome in metazoan [44]. The versatile functions of CTCF generally attribute to a model in which CTCF conformation is a function of differential zinc finger binding to divergent consensus sequences [45, 46]. However, details deserve further study to make the understanding of its function under different conditions more accurate. In a word, our findings showed that circESRP1 and CTCF gene could be designated in suppressive tumor metastasis and progression in RCC, and these might be novel strategies for the treatment of malignant ccRCC.

## Conclusion

Taken together, our research identified the circESRP1 functioned as a suppressor of tumor progression and metastasis in RCC patients. CTCF specifically regulated by circESRP1/miR-3942 pathway could promote the circESRP1 transcript expression. We validated that the overexpression of circESRP1 restrained tumor progression both in vivo and in vitro by the downregulation of c-Myc-mediated EMT pathway via CTCF-dependent positive feedback loop. These findings shed light on the pathogenic mechanism of circESRP1 for ccRCC, providing potential therapeutic targets for ccRCC treatment.

## Supplementary information

Table S1

Figure S1

Figure S2

Figure S3

Figure S4

Figure S5

Supplementary materials

## Data Availability

The datasets used and/or analyzed during the current study are available from the corresponding author on reasonable request.
