# Supplementary material for: CircESRP1 inhibits clear cell renal cell carcinoma progression through the CTCF-mediated positive feedback loop
Source: Cell Death Dis. 2021 Nov 13;12(11):1081. doi: 10.1038/s41419-021-04366-4 (PMC8590696; doi:10.1038/s41419-021-04366-4)
Supplement: Supplementary file 1 — Table S1 [file 41419_2021_4366_MOESM1_ESM.docx]

**Table S1**

Primer Sequences for Real-Time Quantitative RT-PCR

| Primer |  | Nucleotide sequence |
| --- | --- | --- |
| GAPDH | Forward | 5'- AGAAGGCTGGGGCTCATTTG-3' |
|  | Reverse | 5'- AGGGGCCATCCACAGTCTTC-3' |
| U6 | Forward | 5'- AAAGCAAATCATCGGACGACC-3' |
|  | Reverse | 5'- GTACAACACATTGTTTCCTCGGA-3' |
| ESRP1 | Forward | 5'- GCCAAGCTAGGCTCGGATG-3' |
|  | Reverse | 5'- GCCAAGCTAGGCTCGGATG-3' |
| CTCF | Forward | 5'- GTGTTCCATGTGCGATTACG-3' |
|  | Reverse | 5'- TCATGTGCCTTTTCAGCTTG-3' |
| miR-3942-5p | Forward | 5'- GCGCGTAAAGTCCATTGTCAT-3' |
|  | Reverse | 5'- AGTGCAGGGTCCGAGGTATT-3' |
| circESRP1 | Forward | 5'- CACCGAGACCTAGCACTACA-3' |
|  | Reverse | 5'- AAGTTCCATCTTGCTGCACC-3' |
