# Supplementary figures and images for: CircESRP1 inhibits clear cell renal cell carcinoma progression through the CTCF-mediated positive feedback loop
Source: Cell Death Dis. 2021 Nov 13;12(11):1081. doi: 10.1038/s41419-021-04366-4 (PMC8590696; doi:10.1038/s41419-021-04366-4)

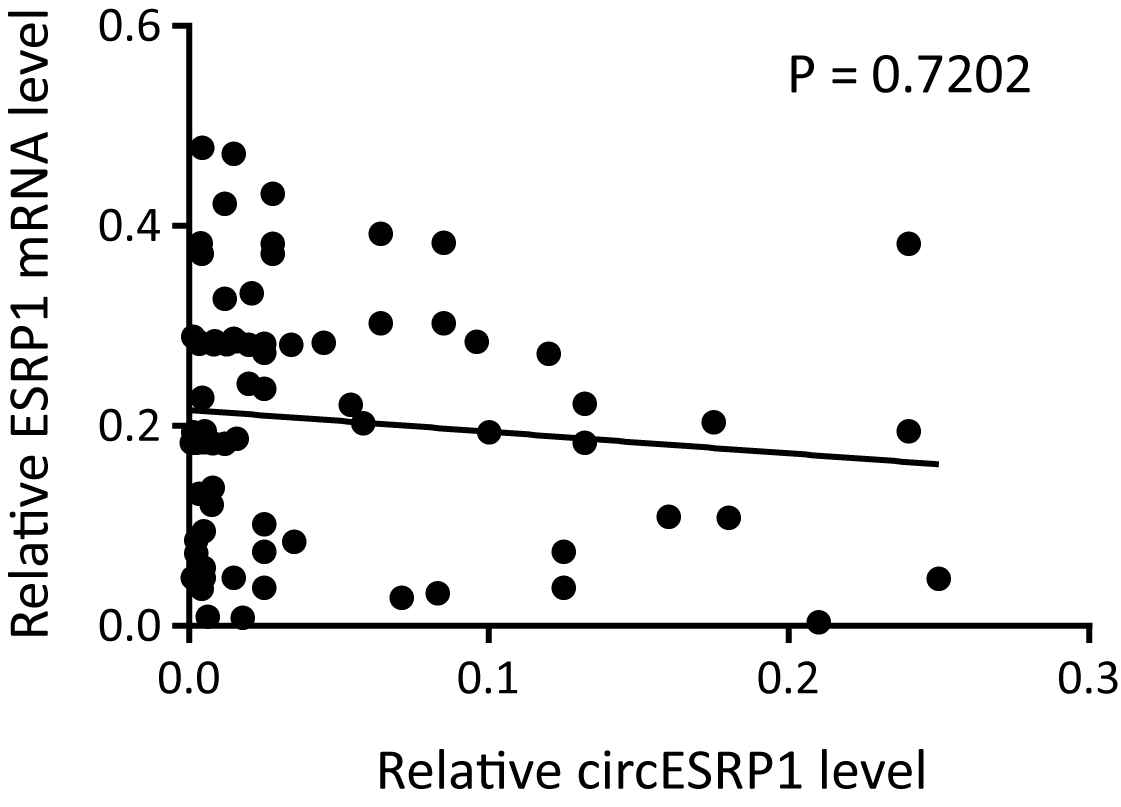

Supplement: Supplementary file 2 — Figure S1 [file 41419_2021_4366_MOESM2_ESM.tif]

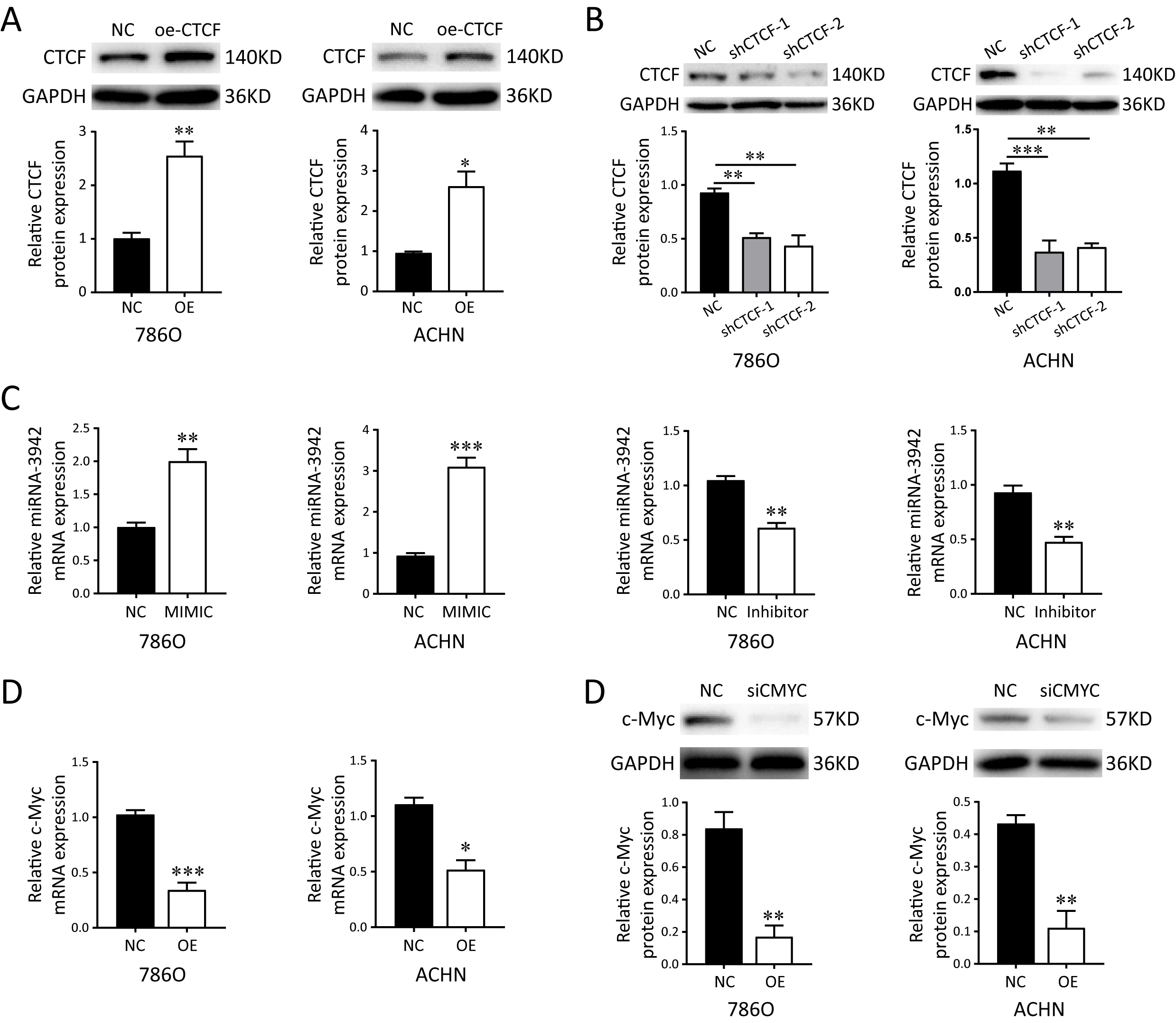

Supplement: Supplementary file 3 — Figure S2 [file 41419_2021_4366_MOESM3_ESM.tif]

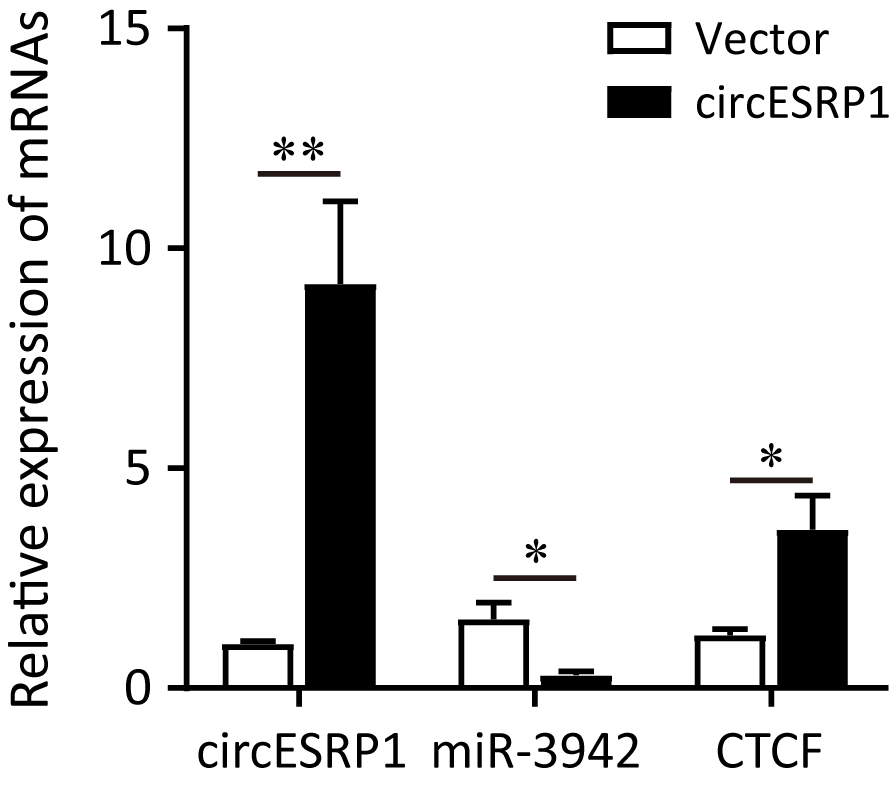

Supplement: Supplementary file 4 — Figure S3 [file 41419_2021_4366_MOESM4_ESM.tif]

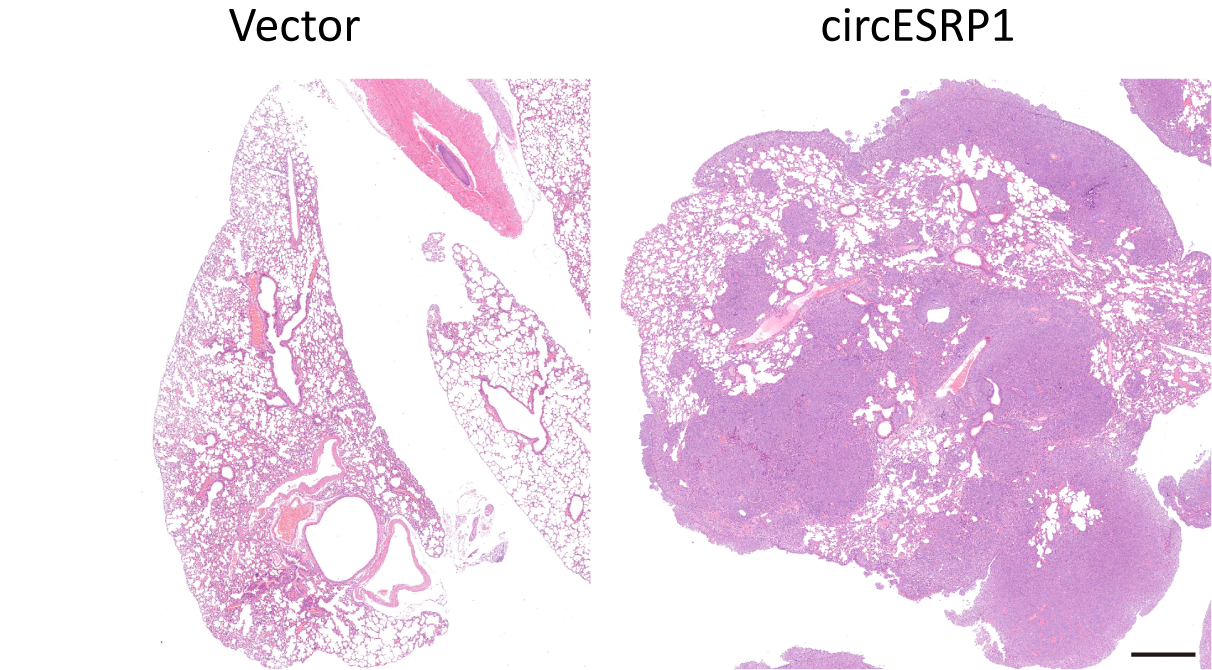

Supplement: Supplementary file 5 — Figure S4 [file 41419_2021_4366_MOESM5_ESM.tif]

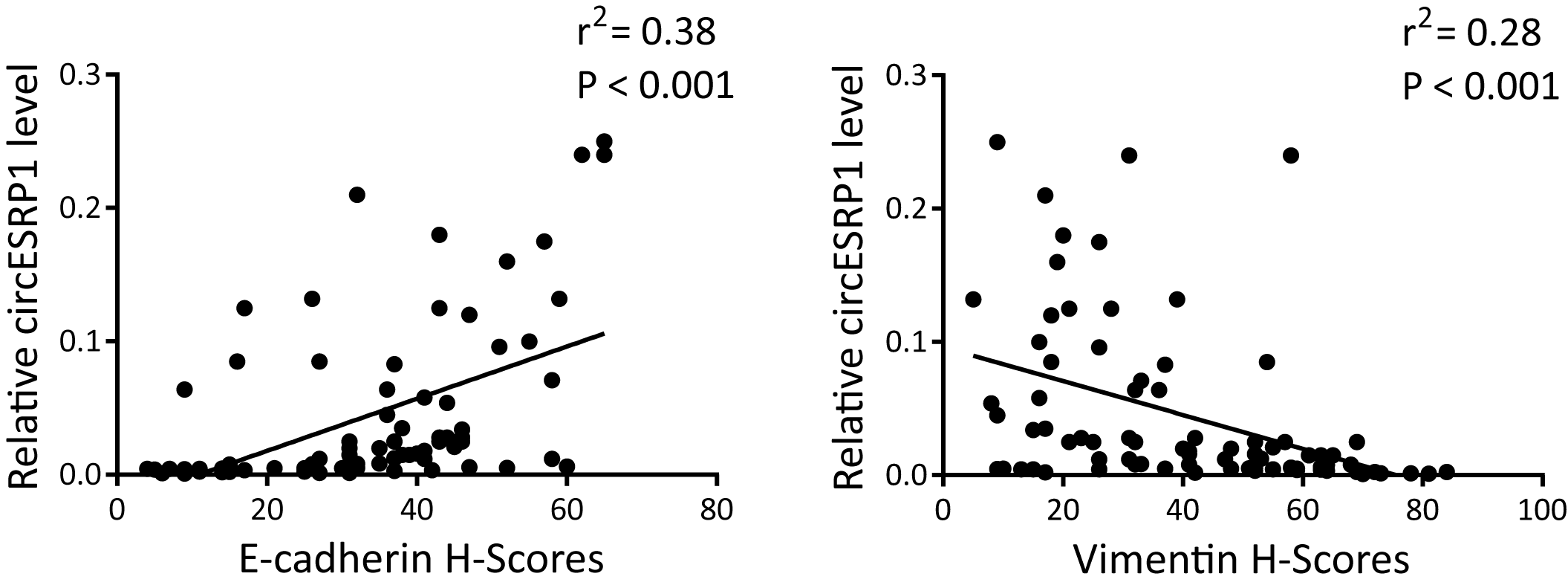

Supplement: Supplementary file 6 — Figure S5 [file 41419_2021_4366_MOESM6_ESM.tif]
