# Supplementary material for: CircESRP1 inhibits clear cell renal cell carcinoma progression through the CTCF-mediated positive feedback loop
Source: Cell Death Dis. 2021 Nov 13;12(11):1081. doi: 10.1038/s41419-021-04366-4 (PMC8590696; doi:10.1038/s41419-021-04366-4)
Supplement: Supplementary file 7 — Supplementary materials [file 41419_2021_4366_MOESM7_ESM.docx]

**Supplementary materials**

**Table S1**

Primer Sequences for Real-Time Quantitative RT-PCR.

**Figure S1**

There was no significant correlation between the expression of ESRP1 linear RNA and circESRP1.

**Figure S2**

The transfection efficiency of CTCF plasmid, c-MYC plasmid, microRNA mimic and inhibitor were verified by western blot or qRT-PCR

**Figure S3**

Relative expression levels of circESRP1, miR-3942 and CTCF were compared in circESRP1 overexpressed and control group in vivo.

**Figure S4**

Representative images of harvested lung lobes with haematoxylin-eosin (H&E) staining of the tumor burden (3 sections per lung). Bar = 500μm.

**Figure S5**

The expression levels of circESRP1 negatively correlated with Vimentin in ccRCC tissues, while it showed a positive correlation with E-cadherin (n = 79).
